# Antenatal embolization of a large placental chorioangioma: a case report

**DOI:** 10.1186/1752-1947-6-183

**Published:** 2012-07-03

**Authors:** Inas Babic, Maha Tulbah, Wesam Kurdi

**Affiliations:** 1 Department of Obstetrics and Gynecology, King Faisal Specialist Hospital and Research Centre, MBC-52, PO Box 3354, Riyadh, 11211, Kingdom of Saudi Arabia

## Abstract

**Introduction:**

A chorioangioma is the most common benign tumor of the placenta. The majority of pregnancies with chorioangiomas are asymptomatic. Pregnancies with large chorioangiomas are associated with maternal and fetal complications, such as growth restriction, cardiomegaly, congestive heart failure, fetal anemia, thrombocytopenia, nonimmune hydrops and intrauterine fetal death. There are several modalities of treatment published to date with various results. Our case was the third such case report published on the successful treatment with antenatal embolization of the feeding vessel of the chorioangioma. To the best of our knowledge, there have been no published cases about antenatal treatment of placental chorioangiomas in Saudi Arabia, or any other Gulf state.

**Case presentation:**

We describe the case of a 28-year-old Arab woman diagnosed at 22 weeks of gestation with a chorioangioma. A glue material - enbucrilate (Histoacryl) - was used for embolization of the feeding vessel. Intrauterine fetal blood transfusions were performed twice, as a treatment for fetal anemia. The fetus developed heart failure at 30 weeks of gestation. A Cesarean section was performed and the outcome was a live baby with right ventricular hypertrophy. The baby was admitted to our neonatal intensive care unit and discharged at 42 days following birth in a stable condition,with follow-up with our cardiology team.

**Conclusion:**

In this case, we found that intrauterine embolization of the feeding vessel of a chorioangioma with Histoacryl was a valid treatment option that carried a small risk considering the good pregnancy outcome.

## Introduction

Chorioangiomas are the most common placental tumors. Generally, if the tumor size is less than 4 cm it does not cause any symptoms during pregnancy, and will often pass undetermined until examination of the placenta following delivery.

Giant chorioangiomas (size > 4 cm) are rare placental tumors associated with a number of adverse effects, including maternal complications and often poor perinatal outcomes. The most common complications are intrauterine growth restriction, fetal anemia, hydrops fetalis and heart failure, with intrauterine fetal death sometimes resulting as a consequence. Several modalities of antenatal treatment for placental chorioangioma have been introduced with various outcomes.

## Case presentation

**Figure 1:**
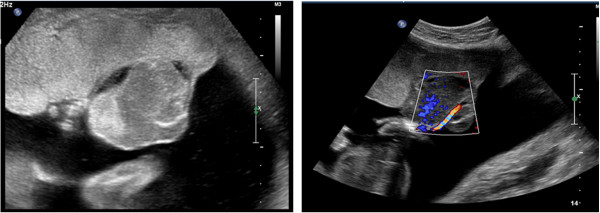
Ultrasound image of the chorioangioma before intervention.

**Figure 2:**
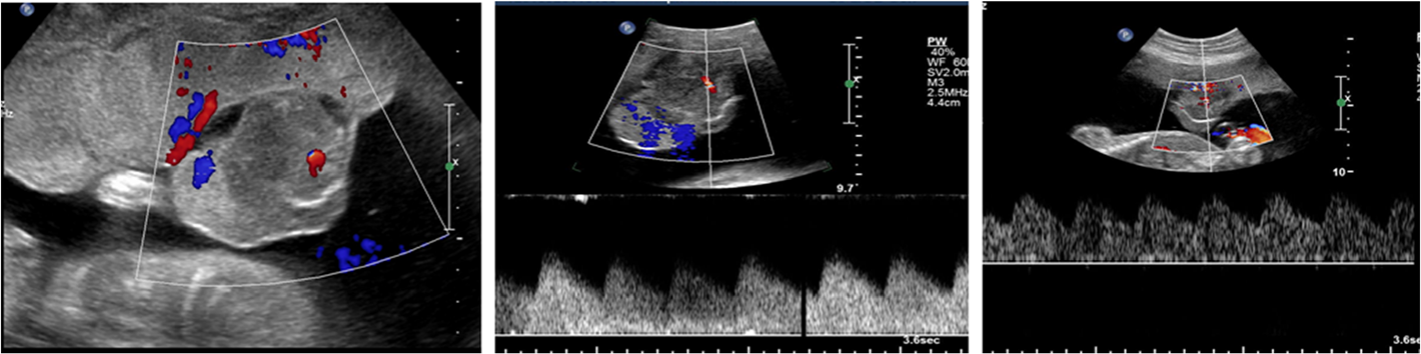
Ultrasound image of the highly vascularized chorioangioma.

Our patient was a 28-year-old Arab woman, gravida 3 para 2 with a history of nonconsanguinity, with two previous full-term normal spontaneous vaginal deliveries and an unremarkable past medical, surgical and family history. She was referred to our hospital at 22 weeks of gestation with severe hydramnios and a placental tumor, for investigations and management. Our patient presented to us complaining of shortness of breath, backache and abdominal pain. An ultrasound examination revealed normal fetal growth for gestational age. Her amniotic fluid index was 48 cm, with the deepest pocket of 13 cm and no signs of fetal hydrops. Ultrasound middle cerebral artery peak systolic velocity (MCA PSV) color Doppler was 48 cm/s, which was 1.71 Multiple of Median (MoM) for gestational age. The placenta was implanted anteriorly with a detectable vascularized tumor measuring 42 mm × 56 mm × 58 mm with a noticeable feeding vessel at the root (Figures 1 and 2).

**Figure 3:**
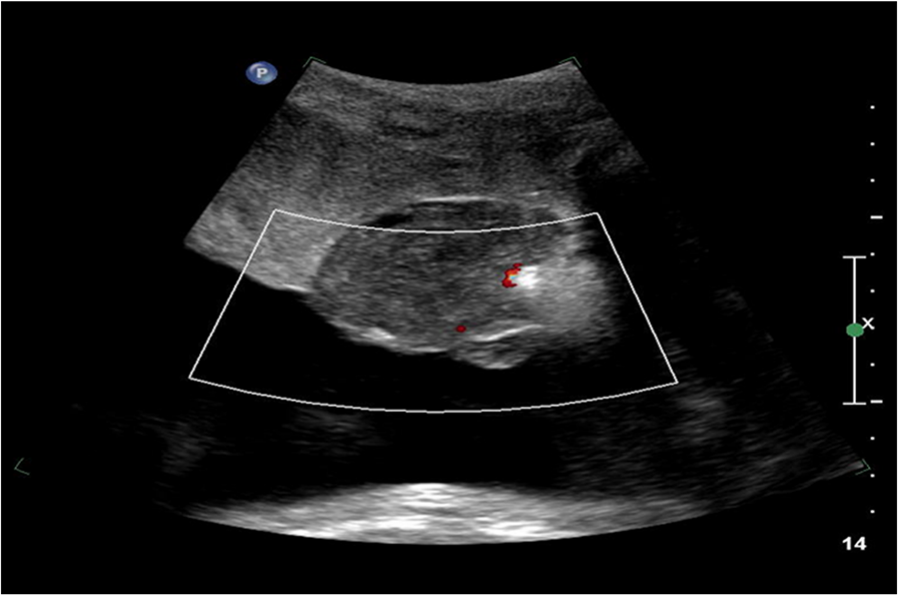
**Chorioangioma post embolization.** Image showing liquid glue, used for embolization of feeding vessel.

After extensive counseling, our patient agreed to undergo percutaneous embolization of the tumor. Employing a multidisciplinary approach involving interventional radiology, we performed an amnioreduction of 3 L for maternal relief of symptoms, followed by injection of 1.5 mL of enbucrilate (Histoacryl; n-butyl-2-cyanoacrylate liquid adhesive glue) diluted with Lipiodol Ultrafluide, at ratio of 1/5, into the feeding vessel of the tumor. After the procedure, ultrasound Doppler revealed a complete absence of flow through the tumor (Figure 3). Cordocentesis was then performed and revealed a hemoglobin level of 10 g/L with hematocrit 28%. The fetus was transfused 50 mL of O-negative blood and the post transfusion hemoglobin level was 14 g/L with hematocrit 42%. Chromosomal analysis revealed a normal female karyotype. Ultrasound was repeated one and four days post procedure and showed a decrease in the amniotic fluid index, of 32.2 cm with a deepest pocket of 10 cm.

**Figure 4:**
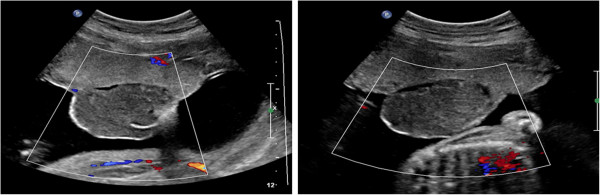
**Chorioangioma post embolization.** Ultrasound images showing no flow through the tumor.

The pregnancy was followed up weekly with ultrasound, which showed normal fetal growth, stable amniotic fluid index with no signs of hydrops, and no flow through the placental tumor (Figure 4).

At 29 weeks’ gestation, ultrasound Doppler of the MCA PSV was 76.3 cm/s. This was 1.97 MoM for gestational age, with the development of mild pericardial effusion. The fetus was transfused with 50 mL of O-negative blood. Pre- and post-transfusion hemoglobin levels were 10.4 g/L and 14.9 g/L, with hematocrit 29% and 44% respectively.

At 30 weeks and 2 days of gestation, an ultrasound detected poor fetal right ventricular contractility with enlarged thick ventricular walls and mild pericardial effusion. Ultrasound Doppler of the MCA PSV was 61.3 cm/s, which was 1.51 MoM for gestational age, and an amniotic fluid index of 19.9 cm. Two intramuscular injections of betamethasone 12 mg were given 24 hours apart and then an elective Cesarean section performed. The outcome was a live female baby weighing 1.6 kg, with Apgar score 5, 7 and 8. No skin lesions or dysmorphic features were seen. The baby was transferred to the neonatal intensive care unit (NICU).

**Figure 5:**
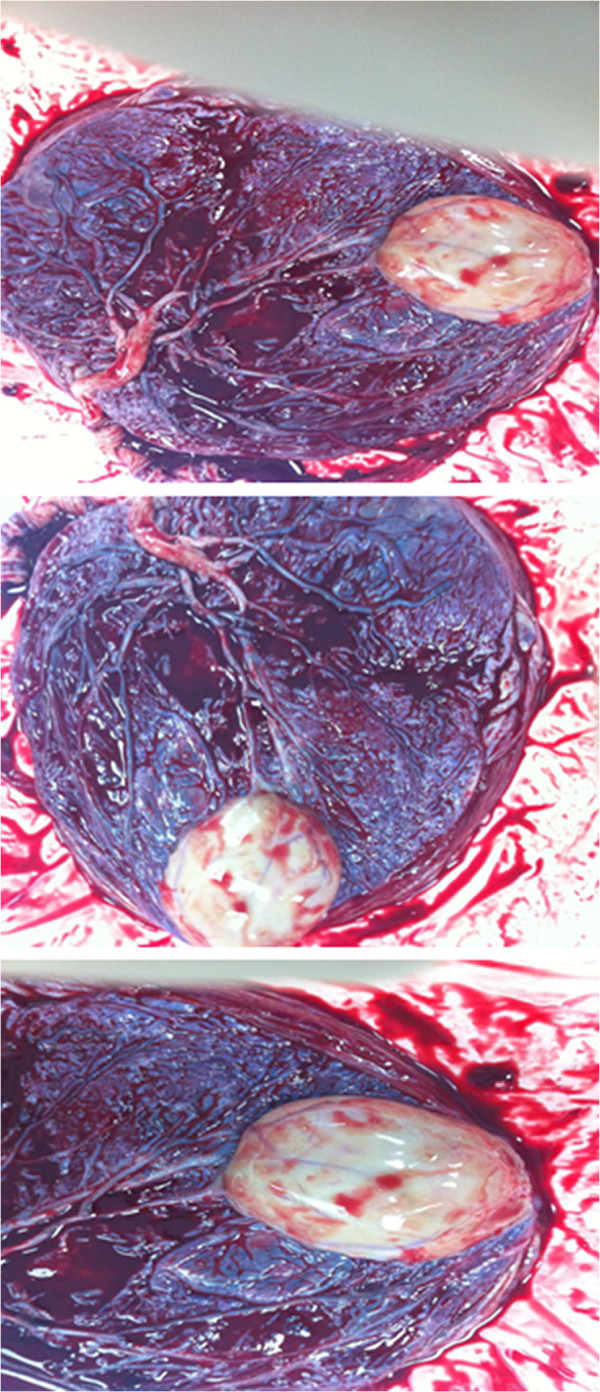
Postpartum gross examination of the placenta.

Gross examination of the placenta revealed a yellowish, well-circumscribed firm mass measuring 5 cm × 5 cm connected by two vessels to the placenta. Histopathologic examination revealed a placental disc 15 cm × 17 cm × 13 cm, with a three-vessel umbilical cord that was attached peripherally and measured 9 cm × 1.5 cm (Figure 5). The weight of the placenta was 530 g. The tumor was confirmed to be a chorioangioma.

Our patient’s baby stayed in the NICU for 42 days. Ultrasound of her head and abdomen showed no abnormalities. Echocardiography confirmed right ventricular hypertrophy with a mildly dilated right ventricle and depressed systolic function. The baby was weaned from the ventilator. Oxygen and nitric oxide were tapered along with inotropic medications. The baby was eventually discharged from the NICU at 42 days old with a follow-up appointment with our cardiology team.

## Discussion

Chorioangiomas are the most common benign tumors of the placenta, with an overall prevalence of 0.9% of pregnancies. Large chorioangiomas (>4 cm) are occurring less frequently, in one out of 9,000 to 50,000 pregnancies. The majority of pregnancies with chorioangiomas are asymptomatic, but pregnancies with large chorioangiomas are associated with maternal and fetal complications, such as growth restriction, cardiomegaly, congestive heart failure, fetal anemia, thrombocytopenia, nonimmune hydrops and intrauterine fetal death [1-4].

In a view of the association between placental chorioangioma and poor pregnancy outcome, close surveillance is recommended. The management of this tumor remains a challenge in fetal therapy practice. If complications develop late in pregnancy, delivery should be strongly considered. Where second or early third trimester complications occur, which are usually more severe, in utero treatment should be considered, after counseling of the patient. There are several modalities of treatment published to date, with various results. These include endoscopic laser coagulation of feeding vessels [5], alcohol injection [6], microcoil embolization [7] and therapeutic amniodrainage [8] . Our case was the third case report published on the successful treatment with antenatal embolization of the feeding vessel of the chorioangioma [9,10]. It is a very simple, safe and effective procedure with no fetal toxicity. It can be performed at any gestational age where facilities for intrauterine therapy are available.

## Conclusion

In this case, we found that intrauterine embolization of the feeding vessel of a chorioangioma with Histoacryl was a valid treatment option that carried a small risk considering the good pregnancy outcome.

## Consent

Written informed consent was obtained from the patient for publication of this case report and any accompanying images. A copy of the written consent is available for review by the Editor-in-Chief of this journal.

## Competing interests

The authors declare that they have no competing interests.

## Authors’ contributions

IB participated in the medical management and wrote the case report. MT carried out the antenatal and postnatal management and supervised the coordination and elaboration of the case report. WK participated in the medical management and supervised the elaboration of the case report. All authors read and approved the final manuscript.
